# Characterization of bone marrow lesions in axial spondyloarthritis using quantitative T1 mapping MRI

**DOI:** 10.1007/s00256-024-04583-w

**Published:** 2024-01-15

**Authors:** Torsten Diekhoff, Dominik Deppe, Denis Poddubnyy, Katharina Ziegeler, Fabian Proft, Felix Radny, Christoph Niedermeier, Kay Geert Hermann, Marcus R. Makowski

**Affiliations:** 1 Department of Radiology, Charité - Universitätsmedizin Berlin, Campus Mitte, Humboldt-Universität zu Berlin, Freie Universität Berlin, Charitéplatz 1, 10117 Berlin, Germany; 2 Department of Gastroenterology, Infectiology and Rheumatology (including Nutrition Medicine), Charité – Universitätsmedizin Berlin, corporate member of Freie Universität Berlin and Humboldt-Universität zu Berlin, Berlin, Germany; 3 Present Address: Institute of Diagnostic and Interventional Radiology, School of Medicine and Health, Technical University of Munich, Munich, 81675 Germany

**Keywords:** Axial spondyloathritis, Magnetic resonance imaging, Quantitative imaging, Active inflammation, Bone marrow edema

## Abstract

**Objective:**

Conventional magnetic resonance imaging (MRI) uses T1-weighted and short-tau inversion recovery (STIR) sequences to characterize bone marrow in axial spondyloarthritis. However, quantification is restricted to estimating the extent of lesions because signal intensities are highly variable both within individuals and across patients and MRI scanners. This study evaluates the performance of quantitative T1 mapping for distinguishing different types of bone marrow lesions of the sacroiliac joints.

**Materials and methods:**

In this prospective study, 62 patients underwent computed tomography (CT) and MRI of the sacroiliac joints including T1, STIR, and T1 mapping. Bone marrow lesions were characterized by three readers and assigned to one of four groups: sclerosis, osteitis, fat lesions, and mixed marrow lesions. Relaxation times on T1 maps were compared using generalized estimating equations and receiver operating characteristics (ROC) analysis.

**Results:**

A total of 119 lesions were selected (sclerosis: 38, osteitis: 27, fat lesions: 40; mixed lesions: 14). T1 maps showed highly significant differences between the lesions with the lowest values for sclerosis (1516±220 ms), followed by osteitis (1909±75 ms), and fat lesions (2391±200 ms); *p*<0.001. T1 mapping differentiated lesions with areas under the ROC curve of 99% (sclerosis vs. osteitis) and 100% (other comparisons).

**Conclusion:**

T1 mapping allows accurate characterization of sclerosis, osteitis, and fat lesions at the sacroiliac joint but only for homogeneous, non-mixed lesions. Thus, further sequence development is needed before implementation in clinical routine.

## Introduction

Bone marrow changes in patients with axial spondyloarthritis (axSpA) include active bone marrow inflammation (osteitis, bone marrow oedema), fatty marrow metaplasia, and sclerosis [1]. To correctly characterize the different lesion types, two separate pulse sequences in magnetic resonance imaging (MRI) are widely accepted as imaging standard: 1^st^, an unenhanced T1-weighted (T1) sequence and, 2^nd^, a fluid-sensitive fat-saturated sequence, typically a short tau inversion recovery (STIR) [2]. On T1-weighted images, fat metaplasia is hyperintense compared with normal bone marrow, while sclerosis and osteitis have lower signal intensity. In contrast, on STIR images, osteitis has a high signal while sclerosis and fat metaplasia are hypointense. Therefore, both pulse sequences are needed for correct lesion differentiation. Notably, MRI is less sensitive for sclerosis compared to X-ray-based imaging including computed tomography (CT) [3].

However, even when both MRI sequences are used, quantitative assessment and comparison of measured signal intensities across patients or scanners are challenging, hampering lesion definition based on mean cutoffs or intra- or interindividual comparison in the setting of scientific studies or when imaging is performed to assess treatment responses [4]. Previously introduced fast quantitative MRI sequences such as T1 mapping allow measurements that can be compared in serial follow-up examinations and across patients [5]. This type of MR sequence has the potential to characterize and categorize lesions with a single sequence, thus, improving standardization and allowing implementation of lesion cutoffs for scientific purposes and clinical practice.

Osteitis shows an increased water content and results in longer T1 relaxation times in T1 mapping sequences. This elongation in relaxation time is attributed to the interactions between water molecules and the extracellular matrix, influencing how quickly protons in water realign to their equilibrium state after the disruption caused by the MRI signal. On the other hand, lesions with heightened fat content are identified by shorter T1 relaxation times on T1 mapping, owing to the distinct magnetic characteristics of fat molecules that facilitate a faster return to equilibrium. Furthermore, in the context of sclerosis, the dense and fibrous nature of the tissue leads to a reduction of T1 relaxation times, modifying the local magnetic environment [6]. T1 mapping’s capacity to yield detailed information about the molecular composition within bone marrow lesions renders it a valuable diagnostic and monitoring tool for various bone conditions.

This study aimed at investigating a T1 mapping sequence to characterize bone marrow lesions of the sacroiliac joint (SIJ) in patients with suspected or known axSpA. Specifically, we tested whether T1 mapping can differentiate and quantify lesions and, thus, has the potential to replace the current clinical MR sequence protocol.

## Methods

The institutional review board approved this prospective study under EA1/086/16. All methods were performed in accordance with the declaration of Helsinki and relevant national and institutional guidelines. All patients gave written informed consent.

### Patients

Patients with suspected or known axSpA were consecutively included in this prospective study. All patients underwent imaging for initial diagnosis or workup of current symptoms. There were no exclusion criteria concerning age, sex, or current therapies.

### Imaging

All patients underwent 1.5 Tesla MRI and computed tomography (CT) on the same day and the same CT (Aquilion One Vision, Canon Medical Systems) and MRI (1.5-Tesla Avanto, Siemens Healthineers), respectively. [7] The MRI was performed in supine position with the in-build spine coil and a 6-channel body phased array coil positioned over the pelvis. The protocol included conventional oblique coronal T1w (TE of 13 ms, TR of 441 ms, 4 mm slice thickness with 10% gap) and STIR sequences (TE of 50 ms, TR of 3780 ms, TI of 145 ms, 4 mm slice thickness with 10% gap) as well as T1 mapping using a commercially available modified look-locker inversion recovery (MOLLI) with the following parameters: TE of 1.67 ms, TR of 1222 ms, TI start at 554 ms, and TI increment of 80 ms with 8 different TIs, bandwidth 980 Hz/Ts, flip angle 35° and ETL 1. The total acquisition time was 12min 15 s. The fitting was conducted on the scanner with the in-built software. All sequences used an in-plane spatial resolution of 1.2 × 0.6 mm. CT was performed in dual-energy technique using the rotate/rotate-principle with sequential acquisition of a 135 kVp and 80 kVp volume scan with 10 cm z-axis coverage, a rotation time of 0.5 s, and standard automated exposure control with an SD value of 12, resulting in a mean CTDIvol of 13.9±11.8 mGy [8]. Measurements were performed on 120 kV-equivalent blended reconstructions with 4 mm slice thickness in oblique coronal orientation. The in-plane resolution was 0.5×0.5 mm.

### Image scoring

Three readers scored the images for bone marrow lesions: a trained research student with one year of experience in image reading and two musculoskeletal specialists with 11 and 20 years of expertise in musculoskeletal imaging. The readers used the established SIMACT scoring system based on a 24-region modification of the Berlin score [3, 9]. It assigns a score of 0 to 2 for sclerosis (0: no sclerosis, 1: questionable or little sclerosis, 2: evident sclerosis >10 mm), and 0–3 for osteitis and fat lesions (0: no lesion, 1: questionable or small lesion, 2: lesion in up to 66% of the region volume, 3: more than 66% of the region volume). Osteitis, fat metaplasia, and sclerosis were deemed present when two or more readers agreed on its presence with a score of 2 or higher in the respective quadrant. Regarding the strengths of the different imaging modalities, MRI was considered the standard of reference for osteitis and fat metaplasia, while CT served as the gold standard for sclerosis.

### Quantitative measurement

A musculoskeletal radiology specialist carefully aligned MRI and CT images using image fusion techniques and multiplanar reformation and quantitatively measured the signal intensities of the different lesions in T1 and STIR, relaxation times in T1 maps and attenuation in Hounsfield units (HU) in CT. The region of interest (ROI) size was standardized to 50 mm^2^ avoiding healthy bone marrow and cortical bone. Up to four lesions were measured per patient and only one lesion of one specific type per joint.

### Statistics

Descriptive statistics are reported for clinical parameters and the different lesions providing mean ± standard deviation. All lesions included were assigned to one of four categories: sclerosis, osteitis, fat lesions, and mixed lesions (i.e., osteitis and fat, osteitis and sclerosis, or fat and sclerosis). Age distribution between genders was analysed using the Mann-Whitney-*U* test. Due to the clustered nature of the data, comparisons of the lesion types (sclerosis, osteitism, fat) were performed with generalized estimating equations with identity link function. Lesion type and bone (ilium/sacrum) were defined as factors. ROC statistics were performed to test the diagnostic performance for differentiation of lesions in a pairwise fashion. All tests considered multiple comparisons and were adjusted accordingly.

## Results

### Patients

Two initially screened patients were excluded because they could not complete MRI due to severe claustrophobia. Therefore, 62 patients (33 male, mean age 40.2 ± 12.7 years) were included. 56.5% (26/46) of the study patients were HLA-B27 positive, and 40% (25/46) showed positive radiography according to the modified New York Criteria. Mean CRP was 10.7 ± 18.5 mg/dl with a BASDAI of 4.4 ± 1.5. Thirty-seven patients were finally diagnosed with axSpA according to the assessment of an expert rheumatologist being aware of all clinical and imaging findings. There was no significant difference of age between male (40.6±11.8) and female (40.0±13.6) patients (*p*=0.89).

### Image scoring

Eighteen of 62 patients (29%) showed no bone marrow lesions. In the other patients, 38 sclerosis, 27 osteitis and 40 fat lesions were identified and 14 mixed lesions.

### Quantitative measurement

**Fig. 1:**
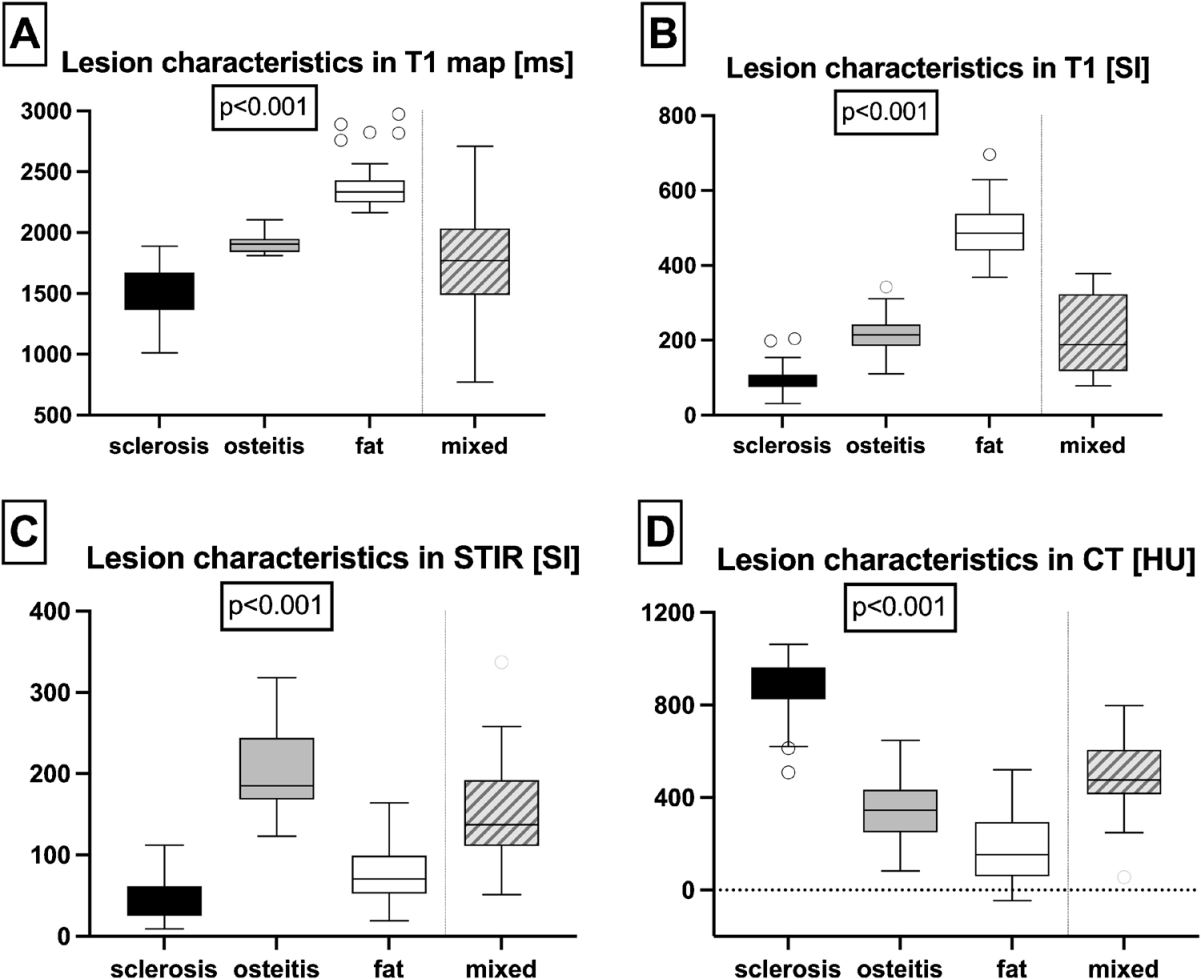
Lesion characteristics in MRI sequences and CT. Presented are the quantitative values measured in the different lesions in T1 mapping (**A**), T1 (**B**), STIR (**C**) and CT (**D**) as Tukey-style box plot: line: mean, box: interquartile range, whiskers 1.5 times the interquartile range (or maximum / minimum value), points: outliers. Values of the T1 map are given in ms, of the CT measurements in HU. Values in B and C are signal intensities of the T1 and STIR respectively. The p-value is derived from generalized estimating equations with identity link function, which was used to test for differences between the three lesion types

**Fig. 2:**
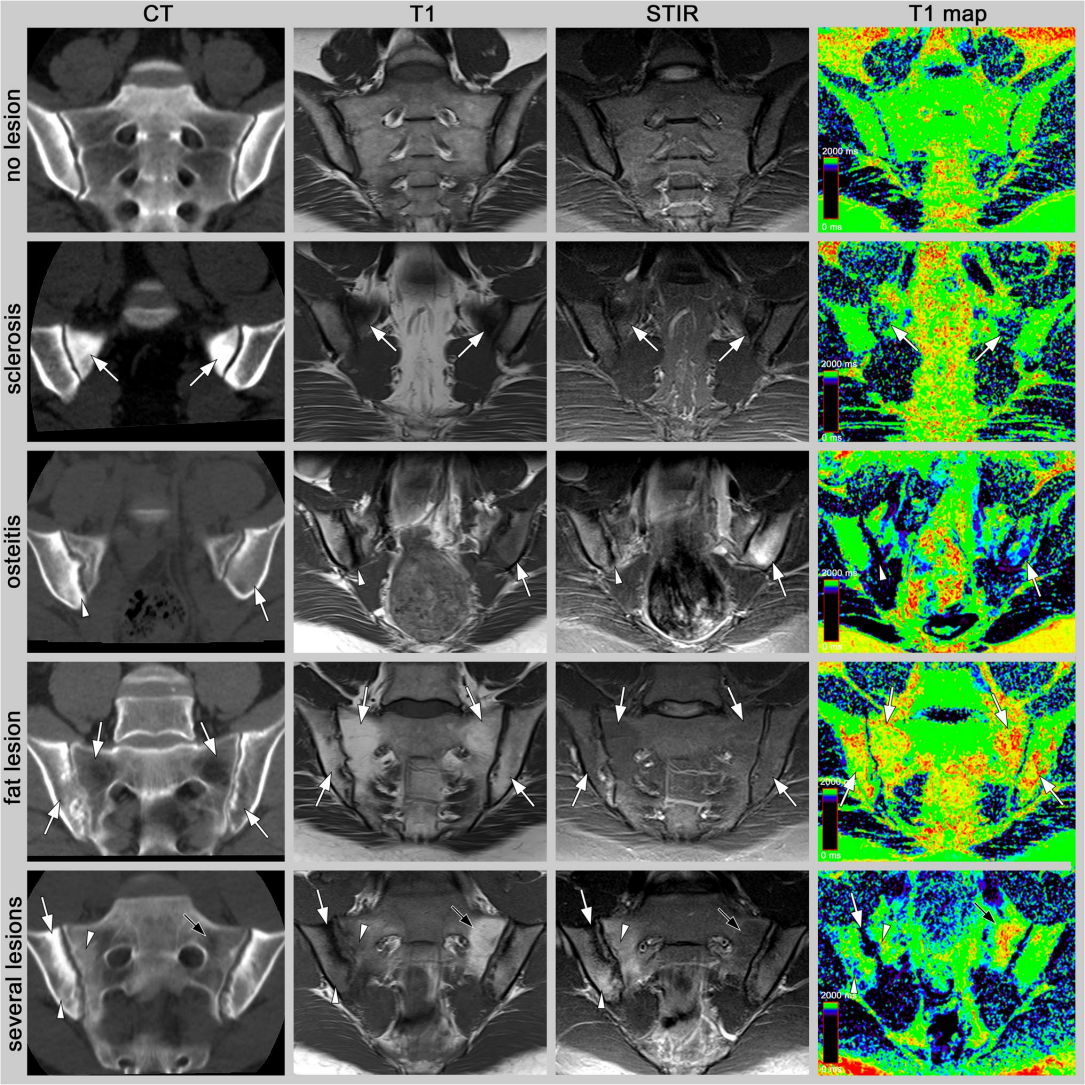
Imaging examples. Presented are different lesions in CT (window level / window width: 240/730) and MRI (T1, STIR, and T1 map). Top row: Normal appearance (no lesions). Sclerosis: A 38-year-old male patient with mechanical joint disease and prominent sclerosis in the anterior aspects of the sacral bone (arrows). Osteitis: An 18-year-old female patient with radiographic axSpA and edema (arrow) and sclerosis (arrowhead). Fat lesion: A 47-year-old male patient with advanced, inactive radiographic axSpA and bilateral fat metaplasia (arrows). Bottom row: A 21-year-old male patient with non-radiographic axSpA (mNYC grading according to radiography 1/1) and sclerosis (white arrow), bone marrow edema (arrowhead), and fat metaplasia (black arrowhead)

**Fig. 3:**
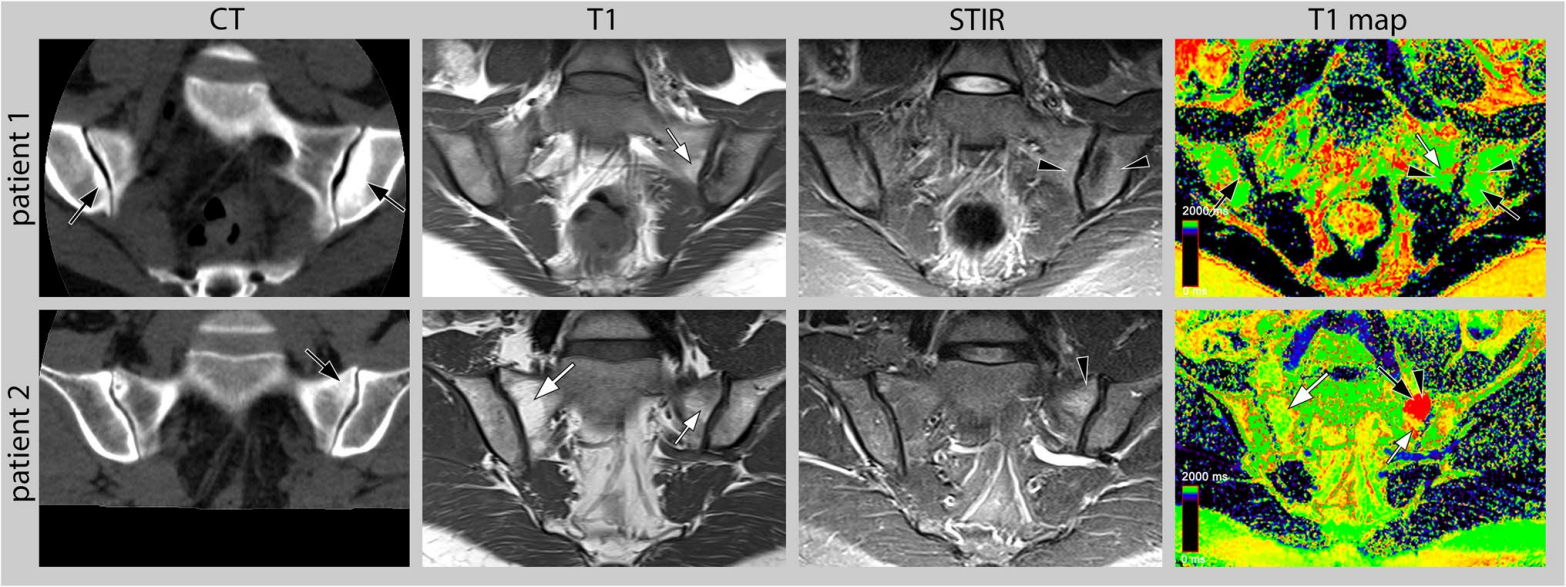
Mixed lesions. Presented are mixed lesions in CT (window level / window width: 240/730) and MRI (T1, STIR, and T1 map). Patient 1 (top row) shows a lesion with sclerosis (black arrow) and bone marrow oedema (black arrowhead) in the left ileum and a lesion with bone marrow oedema and fat (white arrow) in the left sacrum that are not visible in the T1 map. However, the small sclerotic lesions in the right ileum are detectable. Patient 2 (bottom row) shows a mixed lesions of sclerosis (black arrow), fat (white arrow) and bone marrow oedema (black arrowhead) that shows artificially increased values in the T1 map (black arrowhead and arrow). The fat lesion of the right sacrum is displayed normally (white arrow)

Fig. 1 shows the quantitative results for the different lesions. In all pulse sequences, the lesions besides mixed lesions were significantly different in signal intensity in MRI and differed in HU in CT (see also Fig. 2). However, mixed lesions showed a broad distribution of signal intensities irrespective of their imaging characteristics (see Fig. 3). The same was observed for normal bone marrow, which generally showed artificially high values compared to the different lesions due to tissue inhomogeneity.

**Fig. 4:**
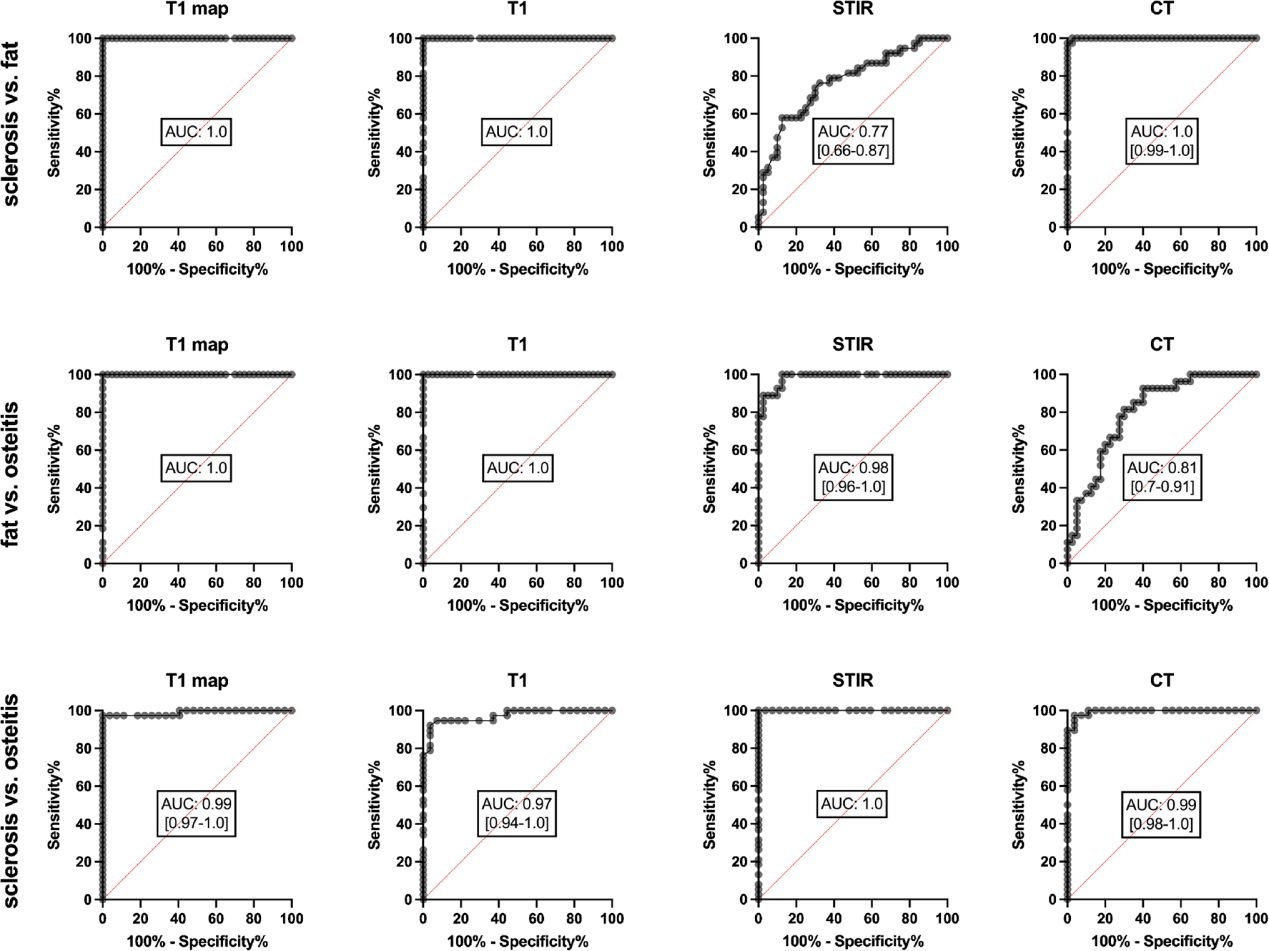
Receiver operating characteristic analysis of T1 maps to differentiate the three different lesion types. AUC: area under the curve. Unsurprisingly, T1 maps and T1 images perform equally well in distinguishing the different lesion types. There is only minimal overlap in sclerosis and osteitis differentiation. STIR performs inferior in differentiating sclerosis and fat while CT is inferior in differentiating osteitis and fat

The results of ROC analysis are presented in Fig. 4. T1w images and T1 maps allowed excellent differentiation of fatty marrow metaplasia from the other lesions. Interestingly, T1w and STIR sequences performed well in distinguishing sclerosis and osteitis with an AUC of 0.97 and 0.99, respectively. Optimal cutoffs were > 2136.5 ms for fat metaplasia and < 1799 ms for sclerosis, resulting in a sensitivity / specificity of 1.0 / 1.0 and 0.97 / 1.0, respectively.

## Discussion

This is the first study using T1 mapping to differentiate bone marrow lesions of the sacroiliac joint. Our results show that T1 mapping accurately distinguishes the three most common bone marrow lesions - osteitis, sclerosis, and fatty marrow metaplasia. Based on our findings, we propose a cutoff of < 1800 ms for sclerosis, 1800 ms to 2150 ms for osteitis, and > 2150 ms for fat metaplasia.

However, inhomogeneity of normal bone marrow resulted in artificially high values in our analysis; therefore, further refinement of the mapping sequences is needed to overcome this disadvantage.

There is a debate whether currently used MRI protocols with a short-tau-inversion recovery sequence are optimal for depicting active bone marrow lesions [10]. Previous studies investigated quantitative MRI for measuring the amount of bone marrow oedema beyond the semiquantitative scoring systems currently in use. Those techniques include diffusion-weighted MRI [11] and T2 mapping [12]; both have the potential to accurately quantify osteitis by assessing diffusion restriction and fluid content, respectively. While both sequences are widely available, diffusion-weighted MRI shows usually low spatial resolution and image quality, while T2 mapping—like T1 mapping—is susceptible to external confounders such as fat fraction. Both would be less suited to quantify non-fluid-containing lesions such as fat lesions or sclerosis. T2 mapping was also applied to the sacroiliac cartilage, which showed increased T2-values in patients with axSpA [13]. The fat fraction calculated from Dixon sequences and R2-values have been investigated to characterize the bone marrow of axSpA patients [14, 15]. Dixon sequences can be performed with high spatial resolution, however, are less sensitive to the properties of the bone marrow compared to the other sequences that were discussed above [16]. Another investigation compared different mapping techniques and found that T1 mapping also allowed quantification of bone marrow oedema to assess the severity of inflammation and the treatment response [17]. Surprisingly, they proved T1 mapping to be superior, for that matter, to the other quantitative MRI techniques. Given the possibilities of better inter-individual comparability, our analysis expands on this by proving that T1 mapping can accurately differentiate between different bone marrow lesions and, therefore, might develop into a one-stop-shop sequence for lesion characterization and quantification, especially for scientific purposes.

The T1-mapping sequence we applied in this study was derived from cardiac applications, where it has been extensively clinically validated [18, 19]. It was also previously successfully applied to the bone [20]. Here, the authors aimed differentiate vital from avital bone after girdlestone arthroplasty. However, while T1-mapping was able to detect bone necrosis with moderate sensitivity and specificity, the authors concluded that there was no added value compared to standard sequences.

In our analysis, we included patients with the diagnosis of axSpA and a variety of different conditions, which might have affected our results and cutoffs. We also did not test for differences between lesion characteristics of axSpA and non-axSpA patients, as the number of patients in each subgroup was deemed too small for such an analysis. Other lesions, such as joint erosion, were beyond the scope of this study. The fat fraction and proton density are a known confounder and might have significantly influenced our mapping results [21]. Our measurements were performed on a 1.5-Tesla machine and the sequence was not tested at 3.0 Tesla by our group. This fact impedes transfer to higher field strengths. Furthermore, no phantom measurements were performed to prove cross-scanner validity. Further refinement of sequence parameters is needed to overcome the limitations of artificially increased signal intensities that we attribute to inhomogeneities of normal marrow composition. Interestingly, this effect helped in identifying pathologic marrow lesions during measurement.

In conclusion, T1 mapping was able to accurately differentiate between different bone marrow lesions based on quantitative measurement in our study. Given its excellent properties for standardization and interindividual comparison and the previously proven ability to quantify active inflammation, T1 mapping might develop into a powerful tool for quantitative bone marrow assessment in axSpA patients for study purposes or future clinical applications. However, in our study, mixed lesions and inhomogeneous bone marrow remained a problem. Thus, further sequence development is needed before implementation in clinical routine.

## Data Availability

The source data are available by contacting the corresponding author with reasonable request.
